# Polysaccharides from Seedless Chestnut Rose (*Rosa sterilis*) Fruits: Insights into Innovative Drying Technologies and Their Structural Characteristics, Antioxidant, Antiglycation, and α-Glucosidase Inhibitory Activities

**DOI:** 10.3390/foods13162483

**Published:** 2024-08-07

**Authors:** Guangjing Chen, Juyan Sun, Qinghua Dai, Meiwen Sun, Peng Hu

**Affiliations:** 1 College of Food Science and Engineering, Guiyang University, Guiyang 550005, China; 17585419235@163.com (J.S.); 17785959192@163.com (Q.D.); 15286718590@163.com (M.S.); 2 School of Pharmacy, Hunan Traditional Chinese Medical College, Zhuzhou 412012, China

**Keywords:** *Rosa sterilis*, polysaccharides, drying method, physicochemical properties, antioxidant, non-enzymatic glycation inhibition activity, hypoglycemic activity

## Abstract

The selection of an optimal drying method is essential for extending the shelf life and enhancing the quality of *Rosa sterilis* fruits. This study investigated the effects of both innovative (microwave vacuum drying and infrared drying) and traditional (freeze-drying and hot air drying) techniques on the structural characteristics and bioactivities of polysaccharides from *R. sterilis* fruits (RSPs). Four different RSPs were obtained from fruits dried using these methods. Results demonstrated that the structural characteristics and bioactivities of RSPs varied significantly with the drying method. Notable differences were observed in extraction yield, total sugar, uronic acid content, monosaccharide molar ratios, molecular weight distribution, particle size, thermal stability, and microstructures of RSPs. Despite these variations, the types of constituent monosaccharides and major glycosidic linkages remained consistent across all methods. Notably, RSPs obtained via microwave vacuum drying (RSPs-MVD) showed a higher uronic acid content and lower molecular weight, and exhibited stronger in vitro antioxidant, α-glucosidase inhibitory, and antiglycation activities. These findings suggest that microwave vacuum drying is an effective pre-drying technique for extracting RSPs, making them suitable as bioactive ingredients in functional foods and pharmaceuticals for managing diabetes mellitus and its complications.

## 1. Introduction

*Rosa sterilis* S. D. Shi (RS), known as the seedless chestnut rose or Golden Cili, is a distinctive variety of *R. roxburghii* Tratt (RRT), notable for its lack of thorns and seeds [1]. This species has become an important commercial crop, primarily cultivated in the karst landscapes of Guizhou Province, with an annual fruit production exceeding 100,000 tons [2]. RS fruits are not only enjoyed fresh seasonally but are also widely processed into juices, beverages, and dried products. Renowned for their nutritional and functional components, RS fruits contain significant levels of ascorbic acid, superoxide dismutase, B vitamins, trace elements, and amino acids [3,4,5]. The fruit’s rich composition of functionally active components, such as polysaccharides, flavonoids, and triterpenes, has sparked increased research interest [6,7,8]. Polysaccharides, in particular, are notable for their potent antioxidant and hypoglycemic effects, as identified in our previous studies [9]. Additionally, these polysaccharides have demonstrated strong prebiotic activity, underscoring their health-promoting potential [6]. Given these findings, polysaccharides extracted from RS fruits hold promise for promising applications in the realms of functional foods and pharmaceuticals, highlighting their significance in health and nutrition research.

The fresh fruit of RRT is often overlooked for direct consumption due to its astringent taste, whereas RS fruit is favored for its sweet flavor, making it more palatable. However, a high moisture content (over 80%) and active respiration make fresh RS fruits prone to deterioration and nutritional degradation [5], underscoring the need for effective drying techniques to enhance shelf life, maintain quality, and facilitate further processing. In food processing, prevalent drying methods include hot air drying (HD), freeze-drying (FD), and vacuum drying (VD). HD evaporates moisture through hot air flow, FD sublimates ice directly into vapor, and VD utilizes lower temperatures to evaporate water due to reduced pressure, which lowers water’s boiling point [10,11]. Traditional drying techniques, however, are often criticized for being time-intensive, energy-consuming, inefficient, and often resulting in inferior product quality [12]. To address these drawbacks, innovative drying technologies such as microwave vacuum drying (MVD) and infrared drying (IRD) have been developed. MVD leverages microwave radiation for quick water molecule evaporation while a vacuum system effectively removes the moisture, ensuring even heating and preventing unnecessary overheating of the produce [13]. IRD employs infrared emitters to transmit energy that is absorbed and converted into heat by the produce [14]. These advanced techniques offer the benefits of reduced drying times, enhanced efficiency, and improved quality of the dried products, presenting promising alternatives to conventional methods for fruit and vegetable preservation [15,16]. Although the impacts of different drying methods on the structural and biological attributes of fruit and vegetable polysaccharides are well established [17,18], the specific effects on polysaccharides from RS fruits (RSPs) have yet to be investigated. This knowledge gap about how various drying techniques influence the structural and physicochemical properties and biological activities of RSPs limits our understanding of the optimal processing approaches for RS fruits. Addressing this gap is crucial for unlocking the full potential of RSPs in functional food and pharmaceutical applications, and it could open up new avenues for research into effective preservation methods that maintain or enhance the health benefits of RS fruits.

Diabetes, a complex metabolic disorder, is profoundly influenced by the role of sugars in generating pro-inflammatory and oxidative advanced glycation end products (AGEs) [19]. The glycation process, critical for protein modification, involves reactions between the carbonyl groups of reducing sugars and the amine groups of amino acids, peptides, or proteins, leading to protein aggregate formation [20]. Furthermore, inhibiting α-glucosidase, a key enzyme in carbohydrate metabolism, can reduce postprandial glucose spikes, offering a new strategy in diabetes management [21]. Therefore, the discovery of new inhibitors that can thwart AGEs’ formation and α-glucosidase activity, coupled with antioxidant properties, is deemed critical for advancing therapeutic and preventative strategies in healthcare. The anti-AGE activity of polysaccharides is significantly determined by their monosaccharide composition and molecular weight distribution [22,23]. Research has shown that the chemical and molecular structures of polysaccharides were susceptible to alteration by various drying methods [24], emphasizing the need to elucidate the anti-AGE characteristics of RSPs processed through different drying techniques. Despite the importance of such studies, research into the anti-AGE activity of RSPs remains scant. Addressing this research gap could unlock new applications for RSPs in food science and health, particularly in developing functional foods and natural therapeutics for diabetes management.

Hence, the objective of this research was to thoroughly investigate the effects of both innovative (MVD and IRD) and traditional (HD and FD) drying methodologies on the structural features and antioxidant, antiglycation, and α-glucosidase inhibitory activities of RSPs. To achieve this, RS fruits were subjected to a range of drying methods, including MVD, IRD, HD, and FD. The study then methodically assessed these diverse drying methods’ ability to inhibit glycation, and their α-glucosidase inhibitory effects in vitro. The insights garnered from this investigation aim to furnish a scientific foundation for selecting the most effective drying techniques to produce high-quality RSPs for use in in functional foods and pharmaceuticals.

## 2. Materials and Methods

### 2.1. Materials and Reagents

Fresh RS fruit samples, in their ripening period, were harvested in October 2022 at Zhenning, Anshun, Guizhou (26°2′49.44″ N, 105°41′59.40″ E). The initial moisture content of the RS fruits was 83.36% on a wet basis. Chemical standards, including standard monosaccharides, 3-phenylphenol, and trifluoroacetic acid (TFA), were obtained from TOKYO Chemical Industry Co., Ltd., Tokyo, Japan. Sodium hydroxide was sourced from Thermo Fisher Co., Waltham, MA, USA, while the pullulan polysaccharide calibration kit was procured from Agilent Co., Santa, Clara, CA, USA. P-nitrobenzene α-d-pyran glucoside (PNPG) was acquired from Aladdin Biochemical Technology Co., Ltd. (Shanghai, China), and α-glucosidase (S10050-100U) from Yeyuan Biotech Co., Ltd. (Shanghai, China). Aminoguanidine and acarbose sugar hydrate were purchased from Shanghai Maclin Biochemical Technology Co., Ltd., Shanghai, China. All other chemicals and reagents used were of analytical grade.

### 2.2. Drying Procedure of RS Fruits

To minimize browning, fresh RS fruits were dried under optimal conditions derived from preliminary experiments. They were prepared by washing and slicing them into 10–15 mm slices before being subjected to various drying methods. The fruits were divided into four groups, each undergoing a different drying method: microwave vacuum drying (MVD), infrared drying (IRD), hot air drying (HD), and freeze-drying (FD). Specifically, for MVD, the process was executed using a commercial microwave vacuum dryer (WBZ-10PLC, manufactured by Guiyang Xinqi Microwave Industry Co., Ltd., Guiyang, China). Initially, 6.0 kg of fresh RS fruits was placed in the drying chamber. The drying commenced once the vacuum reached 0.07 MPa, proceeding through five stages of varying microwave powers and durations: 1000 W for 1.2 h, 500 W for 0.8 h, 0 W for 0.4 h, 300 W for 0.2 h, and finally 0 W for 0.4 h. IRD was performed using an intermittent intermediate wave infrared drying apparatus (DHG-9202-3, from Changzhou Henglong Instrument Factory Co., Ltd., Changzhou, China) set to 1000 W. Here, 4.0 kg of the RS fruit slices were evenly spread as a single layer, achieving a surface temperature of 70 °C. Air flow was maintained at 2 m/s, with the heater set 15 cm from the sample. For HD, a hot air oven (DHG-9240A, by Shanghai Qixin Scientific Instrument Co., Ltd., Shanghai, China) at 2000 W was utilized. The HD protocol for 10.0 kg of RS fruits involved three phases: an initial 6 h at 40 °C, followed by 8 h at 50 °C, and concluding with 34 h at 60 °C; the air velocity was controlled to 3 m/s. The FD process involved placing 6.0 kg of RS fruit slices evenly across five shelves within a vacuum freeze-dryer (BLK-FD-1, Jiangsu BoLaiKe Instrument Co., Ltd., Changzhou, China). This dryer operated at a vacuum of 10 Pa and a temperature setting of −55 °C. The drying procedure was structured into a five-stage schedule; it commenced with a 16 h period at a shelf temperature of 5 °C, followed by a 5 h interval at 10 °C. Subsequently, the shelf temperature was adjusted to 20 °C for another 5 h, increased to 30 °C for an additional 5 h, and concluded with a final 20 h stage at 45 °C. The endpoint for all drying methods was set when the moisture content of the RS fruits fell below 5% on a wet basis. After drying, the treated RS fruits were pulverized and sieved through a 40-mesh screen for further use.

### 2.3. Extraction of Polysaccharides from RS Fruits (RSPs)

The extraction of RSPs was conducted using a modified hot water extraction method, as detailed in our previously published work [9]. Initially, 300 g of RS fruit powder, prepared through various drying methods, was defatted with petroleum ether (1:4, *w*/*v*), followed by 24 h of treatment with 90% ethanol (1:4, *w*/*v*) to remove oils and low molecular weight compounds. Subsequently, 100 g of this defatted powder was mixed with deionized water at a 1:20 material-to-liquid ratio and extracted in water at 70 °C twice for 2 h each, followed by centrifugation at 4000 rpm for 10 min. The extract was concentrated under reduced pressure at 54 °C using a rotary evaporator until it was reduced to one-third of its initial volume. Following concentration, proteins were eliminated using the Sevag method (mixing chloroform with *n*-butanol in a 4:1 ratio), and the solution underwent a decolorization process using HPD100 macroporous resin at a 1:7 ratio of resin to extract volume, agitated in a water bath at 37 °C for 12 h. The clarified filtrate was then mixed with anhydrous ethanol to achieve a final concentration of 80% (*v*/*v*) and was stored overnight at 4 °C. The subsequent precipitate was redissolved in deionized water and dialyzed with a molecular weight cutoff of 8000-14000 Da for 48 h. After freeze-drying, the final polysaccharide extracts from the differently dried RS fruits (MVD, IRD, HD, and FD) were designated as RSPs-MVD, RSPs-IRD, RSPs-HD, and RSPs-FD, respectively.

### 2.4. Structural Characterization of the Four RSPs

#### 2.4.1. Determination of Chemical Composition

The extraction yield was quantified by the weight ratio of the freeze-dried RSPs to the initially dried RS fruit powders. The quantification of chemical composition in RSPs-MVD, RSPs-IRD, RSPs-HD, and RSPs-FD was conducted using specific analytical methods: the phenol-sulfuric acid method for total sugars [25], the *m*-hydroxydiphenyl colorimetric method for uronic acids [26], and Bradford’s method for proteins [27].

#### 2.4.2. Determination of Sugar Composition

To analyze the sugar composition of RSPs-MVD, RSPs-IRD, RSPs-HD, and RSPs-FD, we began by weighing 5 mg of each polysaccharide sample and placing it into a 10 mL ampoule. Each ampoule received 4 mL of 2 M trifluoroacetic acid (TFA), sealed with a flame from an alcohol lamp, and hydrolyzed at 105 °C for 6 h. Post-hydrolysis, TFA was removed via rotary evaporation under reduced pressure, followed by co-distillation with methanol. The dry residues were then redissolved in 50 mL of deionized water and filtered through a 0.22 μm nylon membrane to prepare for chromatographic analysis. Ion chromatography was performed using an ICS 6000 system (Thermo Fisher, Waltham, MA, USA) equipped with a Carbopac PA20 column (3 × 150 mm, 5 μm). A gradient elution program was set as follows: from 0 to 18.0 min, use a solution of 10% NaOH (20 mM) and 90% ultrapure water; from 18.01 to 36.00 min, switch to 10% NaOH (20 mM), 20% sodium acetate (500 mM), and 70% ultrapure water; and from 36.01 to 56.00 min, use a mixture of 50% NaOH (400 mM) and 50% ultrapure water. The flow rate was maintained at 0.3 mL/min, the column temperature at 30 °C, and the injection volume at 25 μL. Monosaccharide components were identified by comparing retention times and analyzing peak area–concentration calibration curves against known standards.

#### 2.4.3. Homogeneity and Molecular Mass Analysis

To assess the homogeneity and molecular weights of RSPs-MVD, RSPs-IRD, RSPs-HD, and RSPs-FD, a high-performance gel-permeation chromatography (HPGPC) system was utilized. The specific model used was the 1290 from Agilent Co., Santa Clara, CA, USA, equipped with a refractive index detector. The chromatographic separation was carried out using a TSK-Gel GMPWXL column (7.8 mm × 300 mm). The elution mobile phase consisted of 0.1 M sodium nitrate (NaNO_3_), with a flow rate of 0.6 mL/min and a column temperature of 30 °C. Molecular weight calibration was performed using a series of pullulan standards with molecular weights (*Mw*) of 667, 6,300, 22,000, 49,700, 216,000, and 334,000 Da to construct the calibration curve. The relationship between the logarithm of molecular weight (Log*Mw*) and retention time (*t*) was established through the regression equation Log*Mw* = −0.5329*t* + 11.741, *R*^2^ = 0.9971.

#### 2.4.4. Fourier Transform Infrared (FTIR) Spectroscopy and Degree of Methyl Esterification (DM) Analysis

For FTIR spectroscopy analysis, 2 mg of RSP powder was finely ground with 300 mg of dried potassium bromide (KBr), then transferred to a compression mold and compacted under vacuum to form a translucent tablet for infrared analysis. The FTIR spectra of the RSP samples were captured using a PE-Spectrum Two spectrometer (Perkin Elmer Co., Waltham, MA, USA). The spectral scan range was set from 4000 to 500 cm^−1^, with a resolution of 4.00 cm^−1^. A blank KBr tablet established the background spectrum for accurate baseline correction. Each RSP sample underwent 64 scans to enhance the signal-to-noise ratio and ensure spectral data reliability. The degree of methyl esterification (DM) was assessed using a modified version of a previously established method [28], recording the peak areas of the methyl-esterified band at 1741 cm^−1^ (*A*_1741_) and the non-esterified band at 1615 cm^−1^ (*A*_1615_). DM was calculated using the formula

(1) $$DM\;(\%)=\frac{A_{1741}}{A_{1741}+A_{1615}}$$

#### 2.4.5. Particle Size and X-ray Diffraction (XRD) Analysis

The particle size and XRD parameters of the different RSPs were assessed using the method detailed in our earlier research [9].

#### 2.4.6. Determination of Thermal Properties

The thermal properties of the different RSPs were evaluated using a differential scanning calorimeter (DSC 4000, Perkin Elmer Co., Waltham, MA, USA). For each analysis, 5.0 mg of RSPs (dry basis) was precisely weighed and placed into a standard aluminum pan, which was immediately sealed to prevent moisture absorption and contamination. An empty aluminum pan was used as the reference for all measurements. The analysis was conducted under a controlled environment with a dynamic inert nitrogen atmosphere, maintaining a flow rate of 50 mL/min. The temperature was gradually increased at a rate of 10 °C/min, starting from 45 °C and extending up to 350 °C.

#### 2.4.7. Scanning Electron Microscopy (SEM) Analysis

The morphology of the four RSP samples was examined using SEM at magnifications of 100× and 20.0 K×, with an operating voltage of 5.0 kV. To prepare for SEM imaging, each sample was sputtered with gold to enhance conductivity. Imaging was performed using a SIGMA 300 SEM (ZEISS, Jena, Germany), which facilitated detailed visualization of the polysaccharides’ microstructural characteristics.

### 2.5. linoleic Acid Peroxidation Inhibition Assay

The antioxidant activities of various RSPs were assessed by quantifying thiobarbituric acid-reacting substances (TBARS) produced during the peroxidation of linoleic acid, utilizing a modified version of a method previously described [29]. For the tests, 1.0 mL of RSPs at concentrations ranging from 0.5 to 8 mg/mL, or ascorbic acid (AA) as a control, was combined with 5.0 mL linoleic acid (40 μM in phosphate buffer, pH 7.0), 5.0 mL phosphate buffer (100 μM, pH 7.4), and 1.0 mL FeSO_4_·7H_2_O (4 mM). Oxidation was initiated by adding 1.0 mL of ascorbic acid (2 mM), and the mixture was then incubated for 24 h at 37 °C. The reaction was halted by introducing 5.0 mL trichloroacetic acid (10%). Subsequently, 2.0 mL this mixture was treated with 2.0 mL thiobarbituric acid (1%) and heated for 10 min at 95 °C. After centrifugation at 4000 rpm for 10 min, the absorbance of TBARS in the supernatant was determined at 532 nm. This protocol assesses the antioxidant activity of RSPs based on their capacity to inhibit lipid peroxidation:

(2) $$Linoleic\;acid\;peroxidation\;inhibition\;(\%)=\frac{A_{c}-A_{s}}{A_{c}-A_{b}}\times \;100$$

where *A*_c_ represents the absorbance of the control, which is the sample without RSPs. *A*_s_ refers to the absorbance of the sample containing RSPs, and *A*_b_ denotes the absorbance of the blank, which lacks both RSPs and FeSO_4_·7H_2_O.

### 2.6. Non-Enzymatic Glycation Activity Assay

#### 2.6.1. Non-Enzymatic Glycation of BSA

The antiglycation efficacy of RSPs was evaluated using the BSA–fructose system, which was adapted from a previously established method [30]. Aminoguanidine (AG) served as a positive control. Solutions containing BSA (20.0 mg/mL) and varying concentrations of RSPs or AG were pre-incubated in PBS (0.1 mM, pH 7.4) for 30 min at 25 °C. Fructose (500 mM) was then added, and the solution was incubated for 24 h at 50 °C. After incubation, the BSA–fructose samples were stored at −20 °C for subsequent analysis.

#### 2.6.2. Analysis of Fructosamine Level

The fructosamine content, indicative of Amadori products in the BSA–fructose system, was measured using the nitro blue tetrazolium (NBT) reduction assay [31]. For this test, 500 μL of the glycated solution was combined with 2.0 mL of 0.3 mM NBT solution and 2.5 mL of sodium carbonate buffer (100 mM, pH 10.35), and the mixture was incubated for 15 min at 25 °C. Absorbance at 530 nm was recorded to calculate the inhibition rate (%) of fructosamine formation using a specified equation:

(3) $$Inhibition\;rate\;(\%)=\frac{Absorbance\;without\;RSPs\;\left(A_{b}\right)-Absorbance\;with\;RSPs\;\left(A_{s}\right)}{Absorbance\;without\;RSPs\;\left(A_{b}\right)}\times \;100$$

#### 2.6.3. Analysis of Dicarbonyl Compounds Level

The concentration of dicarbonyl compounds in the BSA–fructose system was assessed using the Girard-T assay [32]. This method involves reacting the Girard-T reagent with dicarbonyl compounds to produce a product with strong absorption at 290 nm. For the assay, 500 μL of glycated solution was mixed with 50 μL of 500 mM Girard-T reagent and 400 μL of 500 mM sodium formate solution (pH 2.9). The mixture was then incubated for 1 h at 25 °C. The inhibition rate of dicarbonyl compounds was calculated using the same equation as for fructosamine.

#### 2.6.4. Analysis of Fluorescent AGEs

AGE concentrations were determined by their fluorescence in the BSA–fructose system [33]. Specifically, 80 μL of glycated solution was diluted to 2.0 mL with PBS (200 mM, pH 7.4), and fluorescence intensity was measured at excitation/emission wavelengths of 370/440 nm using a spectro-fluorophotometer. The inhibition rates of AGEs were calculated based on the differences in fluorescence intensities between samples with and without RSPs or AG, as follows:

(4) $$Inhibition\;rate\;(\%)=\frac{Fluorescence\;without\;RSPs\;\left(F_{0}\right)-Fluorescence\;with\;RSPs\;\left(F\right)}{Fluorescence\;without\;RSPs\;\left(F_{0}\right)}\times \;100$$

### 2.7. Anti-Postprandial Hyperglycemia Assay In Vitro

#### 2.7.1. Inhibition of α-Glucosidase Activity

α-Glucosidase inhibitory activity was assessed using our established method [9]. Initially, α-glucosidase and PNPG were dissolved in 0.1 mM PBS (pH 6.8). Subsequently, 50 μL of various RSP concentrations were mixed with 50 μL of α-glucosidase solution (0.5 U/mL) and incubated at 37 °C for 10 min. Then, 50 μL of PNPG substrate (5 mM) was added, and the reaction continued for 20 min at 37 °C. Following this, 50 μL of PNPG substrate (5 mM) was added, and the reaction was allowed to continue for another 20 min at 37 °C. The reaction was halted by introducing 200 μL of 1 M Na_2_CO_3_. The inhibitory activity of α-glucosidase was assessed by measuring the release of *p*-nitrophenol from the PNPG substrate at 405 nm. To calculate the α-glucosidase inhibitory rate of RSPs, the following approach was used:

(5) $$Inhibitory\;activity\;\left(\%\right)=\left[1-\left(A_{sample}-A_{blank}\right)/A_{control}\right]\times 100$$

where *A_sample_* represents the absorbance from the reaction mixture containing both RSP samples and α-glucosidase; *A_blank_* is the absorbance of the RSP samples alone, without the enzyme, which helps identify any intrinsic absorbance of the samples; *A_control_* measures the absorbance of the control reaction without RSP samples, indicating the maximum activity of the enzyme. The concentration of RSPs required to inhibit 50% of the enzyme activity (IC_50_) was determined by plotting and analyzing a sigmoidal dose–response curve with a variable slope.

#### 2.7.2. Inhibition Kinetic Analysis

The α-glucosidase inhibitory rate of RSPs was calculated using the following approach, employing the Lineweaver–Burk plot to analyze inhibition kinetics [34]. Kinetic parameters, including *K_m_* (Michaelis constant) and *V_max_* (maximum reaction velocity), were evaluated for α-glucosidase (0.5 U/mL) in the presence of varying concentrations of RSPs (0.5, 2, and 6 mg/mL). Enzyme activity was tested against varying concentrations of PNPG substrate (1 to 4 mM), and the data were plotted on a Lineweaver–Burk graph to ascertain inhibition type and calculate kinetic parameters. The enzyme–inhibitor dissociation constant (*K_i_*) was derived from the plot’s *x*-axis intersection, comparing the slopes of each line to the inhibitor concentrations used. Similarly, the inhibitor–enzyme–substrate dissociation constant (*K_is_*) was determined from the y-axis intercept, correlating each line’s *y*-intercept with the corresponding inhibitor concentration. This approach allowed for a detailed analysis of the inhibition mechanics by RSPs on α-glucosidase.

#### 2.7.3. Fluorescence Spectrum Analysis

To further understand RSP interactions with α-glucosidase, the enzyme’s intrinsic fluorescence was analyzed across various RSP concentrations, slightly modifying our established methodology [9]. Each RSP sample (0.5 to 8 mg/mL) was mixed equally with α-glucosidase solution (0.75 U/mL) and equilibrated for 10 min at 37 °C before fluorescence intensity measurements. Fluorescence was recorded using a spectro-fluorophotometer across 300–500 nm, with excitation set at 280 nm and both excitation and emission bandwidths fixed at 3.0 nm. The analysis of the fluorescent quenching mechanism and the binding parameters were conducted using specific equations:

(6) $$\frac{F_{0}}{F}=1+K_{q}\tau_{0}[Q]=1+K_{SV}[Q]$$

(7) $$\mathrm{lg}\frac{F_{0}-F}{F}=\mathrm{lg}K_{a}+n\mathrm{lg}[Q]$$

The Stern–Volmer equation (Equation (6)) was employed to describe the dynamic fluorescence quenching process, where *F_0_* and *F* represent the fluorescence intensities of α-glucosidase without and with RSPs, respectively. [*Q*] indicates the RSPs concentration, and *τ*_0_ denotes the average lifetime of the fluorescent molecules in the absence of the quencher, assumed here as 10^−8^ s. *K_SV_*, the Stern–Volmer constant, is calculated from the linear regression of the plot of *F*_0_/*F* against [*Q*]. Furthermore, the binding constant (*K_a_*) and the number of binding sites (*n*) were deduced using Equation (7), which facilitates a deeper understanding of the binding affinity and stoichiometry of the interaction between α-glucosidase and RSPs [35].

### 2.8. Statistical Analysis

Data were reported as mean ± standard deviation (SD). The collection of data samples was facilitated using Excel 2021 software (Microsoft, Redmond, WA, USA). These samples were then graphically represented with the assistance of OriginPro 2021 Learning Edition software (Origin Lab Corp., Northampton, MA, USA) and underwent analysis via IBM SPSS Statistics 27.0 software (SPSS Inc., Chicago, IL, USA). Statistical significance was determined using Duncan’s test, with a threshold of *p* < 0.05 indicating significance.

## 3. Results and Discussion

### 3.1. The Yield and Chemical Compositions of RSPs from Different Drying Methods

The extraction yields and chemical compositions of RSPs dried using MVD, IRD, FD, and HD are detailed in Table 1. The yields of RSPs ranged from 4.14% ± 0.03% to 6.93% ± 0.05% and displayed significant differences (*p* < 0.05), consistent with previous findings of yields between 4.36% and 7.11% [9]. Notably, MVD and FD methods significantly enhanced the yields of RSPs, with MVD having a more pronounced effect than FD. Specifically, the extraction yield of RSPs treated with MVD was 39.16% higher than that of the traditional HD method. This increase is likely due to the disruptive effects of high vapor pressure from microwaves, which break down plant cell wall polymers [36]. Additionally, the vacuum conditions in MVD lead to the formation of a puffed structure, improving permeability for extraction agents to penetrate more effectively into plant tissues or cells [36]. FD also resulted in relatively higher yields, possibly due to the creation of a more porous microstructure that facilitates solvent penetration during extraction [17]. This effect may be further enhanced by structural changes in plant matrices caused by ice crystal formation during the freeze-drying process. Conversely, the lower yields associated with HD and IRD could stem from significant fruit shrinkage and the development of a hardened surface (“case hardening”), which occurs during hot air and infrared drying processes [24].

Regarding total sugar content, the polysaccharides followed this order: RSPs-HD < RSPs-IRD < RSPs-FD < RSPs-MVD. RSPs-MVD had the highest total sugar content at 77.07% (*p* < 0.05), closely aligning with previous findings (74.76% to 78.23%) [9]. The four polysaccharides contained minimal protein, ranging from 1.35% to 2.78%, likely due to protein–polysaccharide complexes in the extracts, consistent with previous protein determinations [9]. This suggests that polysaccharides are the primary bioactive components in RSPs. Additionally, the protein and uronic acid levels in RSPs mirrored the neutral sugar content across the four drying methods: 1.49% and 32.48% for HD, 1.35% and 33.76% for IRD, 2.59% and 37.74% for FD, and 2.78% and 41.14% for MVD. The relatively high uronic acid contents in RSPs suggest the presence of pectin-like acidic polysaccharides in seedless chestnut rose fruits. These results indicate a significant impact of drying methods on the main chemical compositions of seedless chestnut rose fruit polysaccharides (*p* < 0.05). The significantly higher levels of neutral sugar, uronic acid, and protein in RSPs-MVD may be due to the activity of enzymes like polysaccharide hydrolase, glucuronidase, and proteolytic enzymes, optimal at 50–80 °C [24]. Microwave vacuum drying, featuring lower temperatures and reduced oxygen concentrations, decreases enzyme activity, resulting in higher retention of neutral sugar, uronic acid, and protein. Moreover, thermal and oxidative processes are crucial [37], explaining the lower retention of these components in RSPs-HD and RSPs-IRD, dried at 60 °C and 70 °C, respectively.

### 3.2. Effects of Different Drying Methods on the Structure Characteristics of RSPs

#### 3.2.1. Molecular Weight Distribution

**Figure 1:**
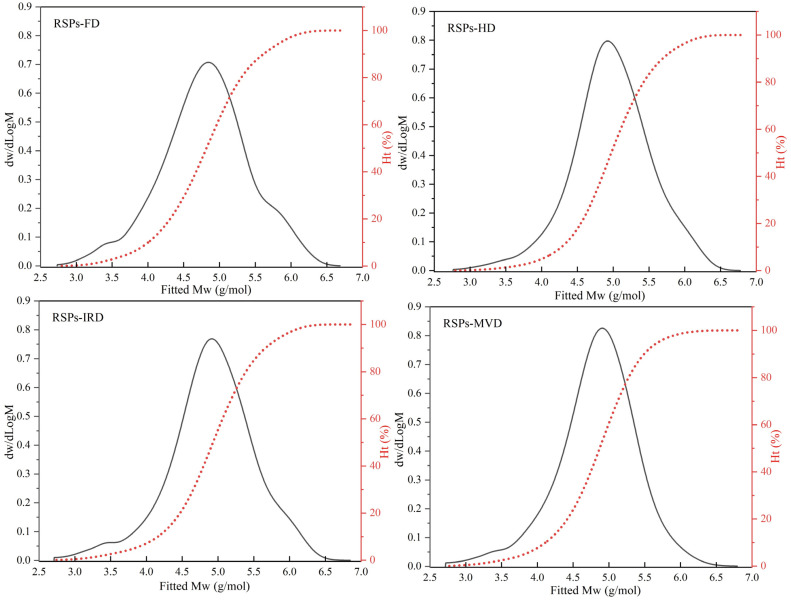
Molecular weight distribution curves of RSPs obtained using different drying methods.

HPGPC analysis revealed the molecular weight distributions of RSPs obtained from various drying methods, as shown in Figure 1. Polysaccharides, being high-molecular-weight polymers, are characterized by number average molecular weight (*Mn*), weight average molecular weight (*Mw*), peak molecular weight (*Mp*), and polydispersity index (*Mw*/*Mn*) [17]. The relevant results for the four RSPs are detailed in Table 1. The findings demonstrate that drying methods significantly affect the molecular weight distributions of RSPs (*p* < 0.05). Specifically, RSPs-HD exhibited the largest Mw at 202.18 kDa, followed by RSPs-IRD at 197.53 kDa, RSPs-FD at 161.64 kDa, and RSPs-MVD at 142.51 kDa. Higher-temperature drying methods like HD and IRD induced aggregation of polysaccharides, resulting in increased *Mw* compared to FD and MVD (*p* < 0.05). During thermal drying (HD and IRD), high temperatures disrupt the hydration layer of polysaccharides, exposing hydroxyl groups and fostering interactions that lead to aggregation. Similar results have been observed in polysaccharides extracted from loquat leaves, indicating that these polysaccharides tend to aggregate when subjected to relatively high drying temperatures [38]. Additionally, microwave treatment degrades polysaccharides through shear forces, causing chain breakage and thermal hydrolysis, which explains the lower *Mw* of RSPs-MVD [39]. FD preserves polysaccharide molecular chains by avoiding high temperatures, thereby preventing thermal degradation and maintaining molecular weight. The polydispersity index reflects the breadth of molecular weight distribution in polysaccharides, with lower values indicating narrower distributions and greater homogeneity. According to Table 1, the *Mw*/*Mn* values for RSPs are as follows: RSPs-FD (8.37) > RSPs-IRD (8.07) > RSPs-HD (5.84) > RSPs-MVD (5.56). RSPs-MVD exhibited the smallest polydispersity index (*p* < 0.05), suggesting better homogeneity, likely due to microwave-induced degradation. Conversely, RSPs-FD showed the highest *Mw*/*Mn*, indicating a wide molecular weight distribution due to minimal thermal aggregation. In contrast, RSPs-HD, experiencing significant aggregation, exhibited a narrower molecular weight distribution. Overall, RSPs-MVD demonstrated the lowest *Mw* and superior homogeneity.

#### 3.2.2. Monosaccharide Composition

**Figure 2:**
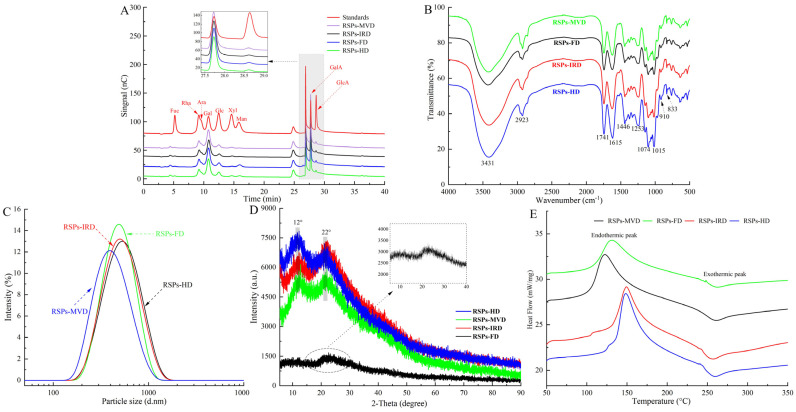
Analytical structure characterizations of RSPs: (**A**) HPAEC chromatograms; (**B**) FT-IR spectra; (**C**) particle size distribution curves; (**D**) XRD spectra; (**E**) DSC thermograms.

High-performance anion-exchange chromatography analysis (Figure 2A) shows that the four RSP samples contain the same types of monosaccharides: fucose (Fuc), rhamnose (Rha), arabinose (Ara), galactose (Gal), glucose (Glc), xylose (Xyl), mannose (Man), galacturonic acid (GalA), and glucuronic acid (GlcA). However, significant variations in their molar ratios suggest that drying methods altered the proportions of these monosaccharides. Notably, Table 2 reveals that RSPs are predominantly composed of Gal and GalA, with a substantial amount of Rha. RSPs-MVD exhibited the highest concentration of galacturonic acid (*p* < 0.05), while RSPs-IRD and RSPs-HD had the lowest, likely due to oxidative degradation from oxygen exposure and elevated temperatures during thermal drying [36]. In contrast, MVD and FD methods better preserved galacturonic acid. Moreover, both methods significantly reduced mannose levels, consistent with previous findings and highlighting the impact of temperature and oxygen on monosaccharide profiles [24]. Pectic polysaccharides are categorized into smooth and hairy regions. The smooth region mainly comprises the homogalacturonan (HG) domain, with partially methylated and acetylated galacturonic acid, while the hairy region includes branched domains such as rhamnogalacturonan-I, rhamnogalacturonan-II, and xylogalacturonan [28]. The content of HG and RG-I can be determined using the formulas “GalA − Rha” and “2Rha + Ara + Gal,” respectively [40]. Pectin linearity, providing insights into polysaccharide structure, is calculated using the formula GalA/(Fuc + Rha + GlcA + Ara + Gal + Xyl), with the percentage of HG reflecting this linearity [41]. According to Table 2, RSPs-MVD showed the highest HG percentage at 22.56%, suggesting greater linearity. All four RSP samples had RG-I percentages over 60%, signifying a rich presence of Ara or Gal side chains. A lower RG-I percentage indicates less branching. Compared to RSPs-HD and RSPs-IRD, RSPs-MVD and RSPs-FD showed more side chains, suggesting better structural preservation. The MR1 ratio (GalA/Rha) reflects the HG-to-RG-I domain proportion, with RSPs-MVD and RSPs-FD displaying higher values (*p* < 0.05), indicative of minimal degradation due to temperature and oxygen. The MR2 ratio “(Ara + Gal)/Rha” measures RG-I branching, indicating average side chain length [42]. RSPs-MVD and RSPs-FD exhibited longer side chains in the RG-I region compared to RSPs-HD and RSPs-IRD, highlighting the effectiveness of MVD and FD in preserving RG-I domain structure.

#### 3.2.3. FT-IR Spectroscopy

The FT-IR spectra of the four RSP samples, displayed in Figure 2B, show that various drying methods did not substantially alter their chemical functional groups. The spectra highlighted strong broad bands from 3600–3200 cm^−1^, indicative of O–H stretching vibrations, and weaker bands from 2900–2800 cm^−1^, linked to C–H stretching vibrations. The absorption peak at 1741 cm^−1^ was identified as the stretching vibrations of esterified carboxylic groups. Moreover, a distinct peak at 1615 cm^−1^ related to the carboxyl groups (-COOR) of galacturonic acid in pectin, verifying the presence of uronic acids [43]. The peak at 1446 cm^−1^ was associated with C-H or O-H vibrations, and the one at 1253 cm^−1^ was tied to C-O-C vibrations, suggesting the presence of -OCH_3_ groups. Notably, typical protein bands at 1651 cm^−1^ and 1555 cm^−1^ were absent, indicating low protein content in the RSPs [44], as detailed in Table 1. Peaks between 1074 cm^−1^ and 1015 cm^−1^, resulting from symmetric vibrations of C–O–C or C–O–H bonds, confirmed the presence of pyran rings [45]. Additionally, peaks around 910 cm^−1^ and 833 cm^−1^ signified *β*- and *α*-configurations, respectively [46]. Further FT-IR spectroscopy analysis evaluated the impact of drying methods on the degree of esterification (DE) of the RSPs. According to Table 2, RSPs-FD showed the highest DE at 58.91% (*p* < 0.05), followed by RSPs-MVD at 56.36%, RSPs-HD at 55.74%, and the lowest DE was noted in RSPs-IRD at 54.68%. The lower DE in RSPs-HD and RSPs-IRD may be attributed to high temperatures during drying, potentially activating pectic esterases and leading to pectin demethylation [39]. With DE values above 50%, all extracted RSPs qualify as high methoxyl pectins, resistant to degradation by endo-α-1,4-polygalacturonase, and stable at high temperatures, ideal for various food processing applications [47]. In conclusion, while the drying methods preserved the structural integrity of the RSPs, they did lead to certain molecular changes, particularly in terms of DE.

#### 3.2.4. Particle Size and Crystal Structure Analysis

Particle size reflects the aggregation level of polysaccharide molecules and can serve as an indirect measure of molecular weight. Figure 2C shows that all four RSP samples display a broad range and a singular peak, indicating a relatively uniform particle size distribution. According to Table 3, the average particle sizes of RSPs-HD and RSPs-IRD were comparable (*p* > 0.05), while RSPs-MVD had a significantly smaller size (p < 0.05), likely reflecting its lower molecular weight, consistent with molecular weight distribution findings. Previous research indicates that polysaccharides with higher molecular weights generally exhibit larger particle sizes [48], explaining the larger sizes observed in RSPs-HD and RSPs-IRD. XRD analysis is a pivotal method for examining the phase and crystal structures of materials. While XRD studies (shown in Figure 2D) verify that RSPs are predominantly amorphous, the diffraction peaks indicate that RSPs-FD has significantly lower intensity compared to the other three polysaccharide samples. This suggests that, although overall crystallinity remains low, it tends to increase under thermal drying conditions. This phenomenon may result from high-temperature drying methods promoting faster aggregation and better organization of the polysaccharide chains, thereby enhancing their crystallinity, a conclusion that aligns with research by An et al. [24].

#### 3.2.5. Thermal Properties

A DSC was employed to examine the thermal behavior of four RSPs across a temperature spectrum of 50–350 °C. As shown in Figure 2E, both endothermic and exothermic peaks were evident in the DSC thermograms for each RSP sample. Table 3 presents comparative data on parameters such as melting temperature (*Tm*), melting enthalpy (Δ*Hm*), degradation temperature (*Tg*), and degradation enthalpy (Δ*Hg*). The *Tm* and *Tg* values for the RSPs spanned 134.03–149.31 °C and 254.96–259.98 °C, respectively. The peak around 130 °C is likely attributable to water evaporation, a process that becomes more pronounced with rising temperatures, facilitating enhanced water absorption as water molecules become more dynamic and migrate to the particle surface [49]. The melting temperatures for RSPs-HD and RSPs-IRD, exceeding 145 °C, suggest a more stable ^4^C_1_ chair conformation of the galacturonic acid ring, indicative of significant transitional stability [47]. As shown in Table 3, the *Tg* and Δ*H*g values for RSPs-MVD and RSPs-FD are significantly higher (*p* < 0.05), indicating a broader melting range and superior thermal stability in these polysaccharides. This enhanced stability may be due to higher branching side chains, such as the (Gal+Ara)/Rha and RG-I ratios (as detailed in Table 2), which are believed to bolster thermal resilience. The observed variances among the four RSPs underscore the significant influence of different drying methods on their thermal properties.

#### 3.2.6. SEM Analysis

**Figure 3:**
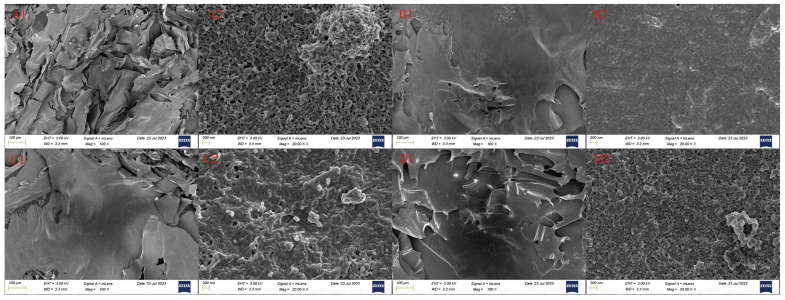
Scanning electron micrographs of RSPs: RSPs-FD (**A**), RSPs-HD (**B**), RSPs-IRD (**C**), and RSPs-MVD (**D**). Each sample is shown at two magnifications: 100× (1) and 20.0 k× (2).

Figure 3 illustrates the significant impact of different drying methods on the microstructure of RSPs. As shown in Figure 3A1–D1, the microstructures of RSPs subjected to IRD and HD are characterized by dense, irregular, massive forms. In contrast, FD and MVD produced flake-like structures with smooth surfaces, characterized by smaller and more uniform particle distributions. At a magnification of 20.0 K×, RSPs-IRD and RSPs-HD exhibited a denser microstructure. The heat-driven process of HD causes the microstructure to shrink and tighten due to temperature gradients and moisture movement. IRD involves the rapid penetration of infrared energy, causing moisture accumulation inside the structure rather than rapid evaporation. This process facilitates crosslinking within RSPs-IRD, resulting in a dense structure with irregular protrusions. In the FD method, water is removed through sublimation, resulting in RSPs-FD having a loose and porous structure. Meanwhile, the MVD process combines the effects of vacuum and microwave drying, producing a porous structure in RSPs-MVD with minimal aggregation.

### 3.3. Antioxidant Activity in a Linoleic Acid System of RSPs from Different drying Methods

**Figure 4:**
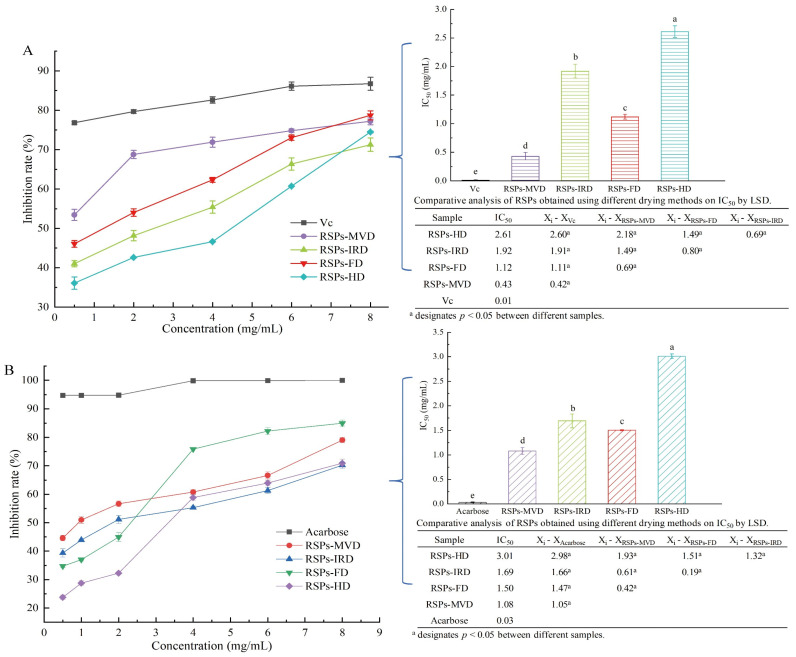

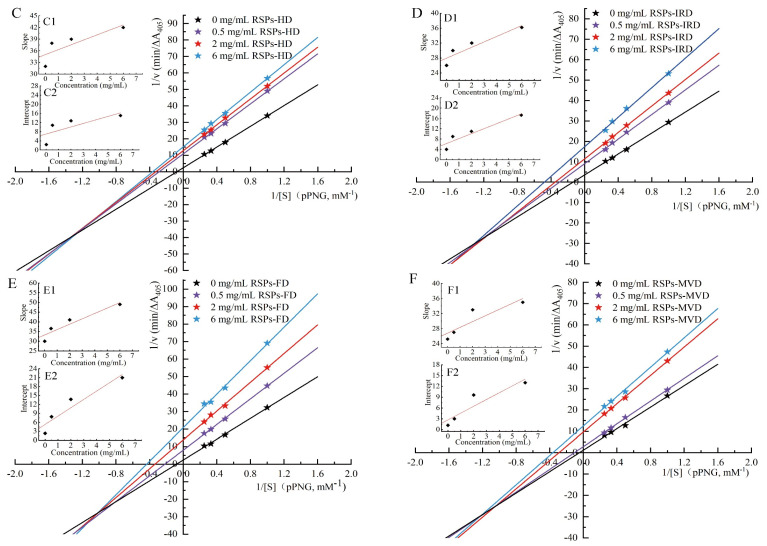
Inhibitory activities and kinetic analyses of RSPs: (**A**) anti-linoleic acid oxidation inhibitory activity; (**B**) α-glucosidase inhibitory activity; (**C**–**F**) Lineweaver–Burk plots of α-glucosidase reactions; (**C1**–**F1**) slope of Lineweaver–Burk plot versus RSP concentration; (**C2**–**F2**) y-intercept of Lineweaver–Burk plot versus RSPs concentration. IC_50_ values with no common letters are significantly different (*p* < 0.05).

In the linoleic acid assay, linoleic acid is susceptible to rancidity, breaking down into various compounds including aldehydes and acids. A key marker of this process is the formation of malondialdehyde (MDA), a three-carbon dialdehyde resulting from oil oxidation, which serves as a primary indicator for assessing lipid oxidation. According to Figure 4A, the antioxidant efficacy of RSPs in a linoleic acid system increases with higher concentrations across all four samples, displaying a dose–response relationship. Among the samples tested, RSPs-MVD showed the most effective inhibition, with an IC_50_ value of 0.43 ± 0.07 mg/mL (*p* < 0.05). This was followed by RSPs-FD with an IC_50_ of 1.12 ± 0.04 mg/mL, RSPs-IRD at 1.92 ± 0.12 mg/mL, and RSPs-HD at 2.61 ± 0.10 mg/mL. However, none matched the potency of the positive control, vitamin C (Vc), which had an IC_50_ of 0.01 mg/mL. These results suggest that RSPs-MVD exhibits the strongest potential for antioxidant resistance in the linoleic acid system among the samples.

### 3.4. In Vitro Hypoglycemic Activity of RSPs from Different Drying Methods

#### 3.4.1. Inhibitory Activity on α-Glucosidase

After consumption, polysaccharides can significantly influence health outcomes through interactions with digestive enzymes, playing a crucial role in modulating blood glucose levels and enhancing insulin sensitivity. One effective strategy for managing postprandial hyperglycemia in type 2 diabetes is to delay glucose absorption by inhibiting carbohydrate hydrolases like α-glucosidase [30]. Consequently, the α-glucosidase inhibition assay is a straightforward and practical method for assessing the hypoglycemic activity of polysaccharides in vitro. According to Figure 4B, both acarbose and the four tested RSPs demonstrated α-glucosidase inhibitory effects that increased with concentration. Among these, RSPs-MVD exhibited significantly higher α-glucosidase inhibitory activity compared to the other RSPs (*p* < 0.05). The IC_50_ values were as follows: RSPs-MVD at 1.08 mg/mL, RSPs-FD at 1.50 mg/mL, RSPs-IRD at 1.69 mg/mL, and RSPs-HD at 3.01 mg/mL. The enhanced effectiveness of RSPs with lower molecular weights and higher uronic acid contents was consistent with our previous findings [9]. The hydroxyl (-OH) and carboxyl (-COOH) groups on the branched chains of polysaccharides can form strong hydrogen bonds with the residues of α-glucosidase, effectively inhibiting its activity. Thus, polysaccharides with higher uronic acid contents and lower molecular weights are considered more effective due to their increased interaction with the enzyme’s active sites [50,51].

#### 3.4.2. Inhibitory Kinetics Analysis

Kinetic studies were conducted to elucidate the mechanisms of enzyme inhibition by RSPs. The findings, illustrated in Figure 4C–F, utilized Lineweaver–Burk double reciprocal plots where different concentrations of inhibitors intersect in the third quadrant. This specific intersection pattern indicates a reduction in both the maximum velocity (*Vmax*) and the Michaelis constant (*Km*) upon the addition of RSPs. The reduction in *Vmax* and *Km* suggests that all four RSPs exhibit mixed-type inhibition. In mixed-type inhibition, the substrate and the inhibitors (RSPs) bind to different sites on the enzyme α-glucosidase. This means that the RSPs do not directly compete with the substrate for the active site. Instead, they bind to alternate sites on the enzyme, which modifies the enzyme’s structure and reduces its catalytic efficiency. This mechanism aligns with findings from previous research. To determine the dissociation constant (*Ki*) of the enzyme–inhibitor complex, one must analyze the *x*-intercept of the Lineweaver–Burk plots [52], which reflects the effect of inhibitor concentration on the slope (shown in Figure 4C1–F1). The dissociation constant for the enzyme–substrate–inhibitor complex (*Kis*) is derived from analyzing the *y*-intercept under varying inhibitor concentrations [53] (shown in Figure 4C2–F2). From the analyses detailed in Table 4, it was found that the *Ki* values for all RSPs are much higher than their *Kis* values. This suggests a stronger binding affinity of the α-glucosidase–PNPG complex (where PNPG is the substrate) with the inhibitors compared to the enzyme alone. Typically, lower *Ki* and *Kis* values signify more potent interactions within both the inhibitor–enzyme–substrate and inhibitor–enzyme complexes, indicating more effective inhibition [54]. Among the tested RSPs, RSPs-MVD displayed lower *Ki* and *Kis* values, indicating the strongest binding affinity to α-glucosidase and confirming its superior inhibitory performance. This observation aligns with the lowest IC_50_ value for RSPs-MVD, underscoring its potent inhibitory effect on α-glucosidase. These findings support the efficacy of this polysaccharide in potentially regulating postprandial blood glucose levels, emphasizing its therapeutic potential in diabetes management.

#### 3.4.3. The Effects of RSPs on Fluorescent Characteristics of α-Glucosidase

**Figure 5:**
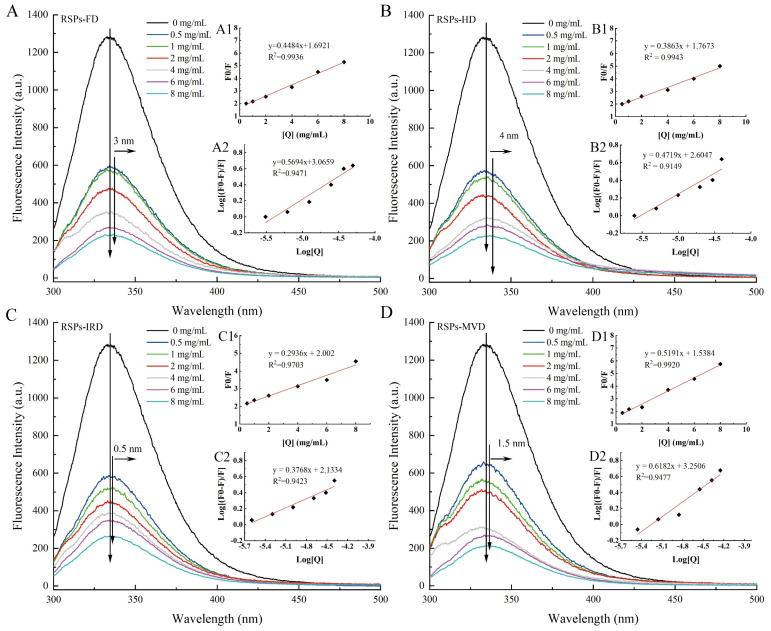
Interaction of α-glucosidase with varying RSP concentrations: (**A**–**D**) fluorescence spectra; (**A1**–**D1**) Stern–Volmer plots; (**A2**–**D2**) double logarithm regression plots of log [(*F*_0_ − *F*)/*F*] against log [*Q*].

The interaction between α-glucosidase and RSPs was explored using fluorescence quenching techniques to identify changes in molecular interactions. Fluorescence quenching involves the reduction of fluorescence quantum yield due to the interaction between a fluorophore and a quencher. As shown in Figure 5A–D, the fluorescence intensity of α-glucosidase decreased as the concentration of RSPs increased. Notably, RSPs-MVD demonstrated the most pronounced quenching effect on α-glucosidase. Tryptophan residues are primarily responsible for the intrinsic fluorescence of α-glucosidase [55]. The slight red shift in the maximum absorption peak for all four RSPs suggests a more hydrophobic microenvironment around the tryptophan residues [56]. This change implies that the tryptophan residues become more exposed, potentially leading to partial unfolding of the α-glucosidase protein structure. Such structural modifications could significantly impact enzyme activity and indicate complex interactions between RSPs and α-glucosidase.

The Stern–Volmer plot is a vital tool in fluorescence spectroscopy used to evaluate the quenching mechanism of fluorophores by quenchers. Figure 5A1–D1 illustrates the Stern–Volmer plots for interactions between α-glucosidase and the four RSPs, with each curve displaying a linear fit and high regression coefficient (*R*^2^ > 0.97). This high linearity indicates reliable interactions between the enzyme and the polysaccharides. From these plots, the Stern–Volmer constant (*K_SV_*) values for RSPs-MVD, RSPs-IRD, RSPs-FD, and RSPs-HD are determined as 7.40 × 10^4^, 5.80 × 10^4^, 7.23 × 10^4^, and 7.81 × 10^4^ M^−1^, respectively (Table 4). These constants reflect the quenching efficiency and binding affinity between the RSPs and α-glucosidase. The corresponding rate constants for dynamic quenching (*Kq*) calculated are 7.40 × 10^12^, 5.80 × 10^12^, 7.23 × 10^12^, and 7.81 × 10^12^ M^−1^ s^−1^ (Table 4). These *Kq* values significantly exceed the theoretical maximum for dynamic quenching (2.0 × 10^10^ M^−1^ s^−1^), suggesting that the observed quenching mechanism is not dynamic but static [57]. Static quenching occurs when the quencher and fluorophore form a non-fluorescent complex before excitation, consistent with the data indicating a predominant static quenching mechanism for all RSPs. This suggests stable complex formation between RSPs and α-glucosidase, further analyzed through the association constant (*Ka*). *Ka* relates to the forming efficiency of the inhibitor–α-glucosidase complex [58]. The *Ka* values, derived from Figure 5A2–D2, for RSPs-MVD, RSPs-IRD, RSPs-FD, and RSPs-HD are 1.78 × 10^3^, 0.14 × 10^3^, 1.16 × 10^3^, and 0.40 × 10^3^ M^−1^, respectively, indicating that RSPs-MVD has the highest binding efficiency. This higher efficiency might be attributed to the lower molecular weight of RSPs-MVD, facilitating a more effective interaction with α-glucosidase. This interaction efficiency likely contributes to the strong inhibitory activity observed for RSPs-MVD in α-glucosidase inhibition assays.

### 3.5. In Vitro Inhibition of Non-Enzymatic Glycation of RSPs from Different Drying Methods

Chronic diabetes often leads to the formation of non-enzymatic AGEs, contributing to complications such as microangiopathy, nephropathy, and atherosclerosis [59]. Glycation begins with the formation of a Schiff base, a reversible intermediate formed when amino groups in proteins react with carbonyl groups from reducing sugars. This Schiff base undergoes the Amadori rearrangement to produce more stable products, participating in the Maillard reaction and eventually forming harmful AGEs through dehydration, oxidation, and further rearrangement [60]. AGEs bind to receptors for AGEs (RAGEs), promoting reactive oxygen species (ROS) production and triggering inflammatory responses, exacerbating oxidative damage and activating pro-inflammatory mediators [61]. Thus, targeting and inhibiting AGE formation is a viable approach to mitigating diabetic complications. This study evaluated the inhibitory effects of four RSPs on non-enzymatic protein glycation using a BSA–fructose model, with aminoguanidine (AG) as the positive control. The glycation process is categorized into three stages: early, intermediate, and late.

**Figure 6:**
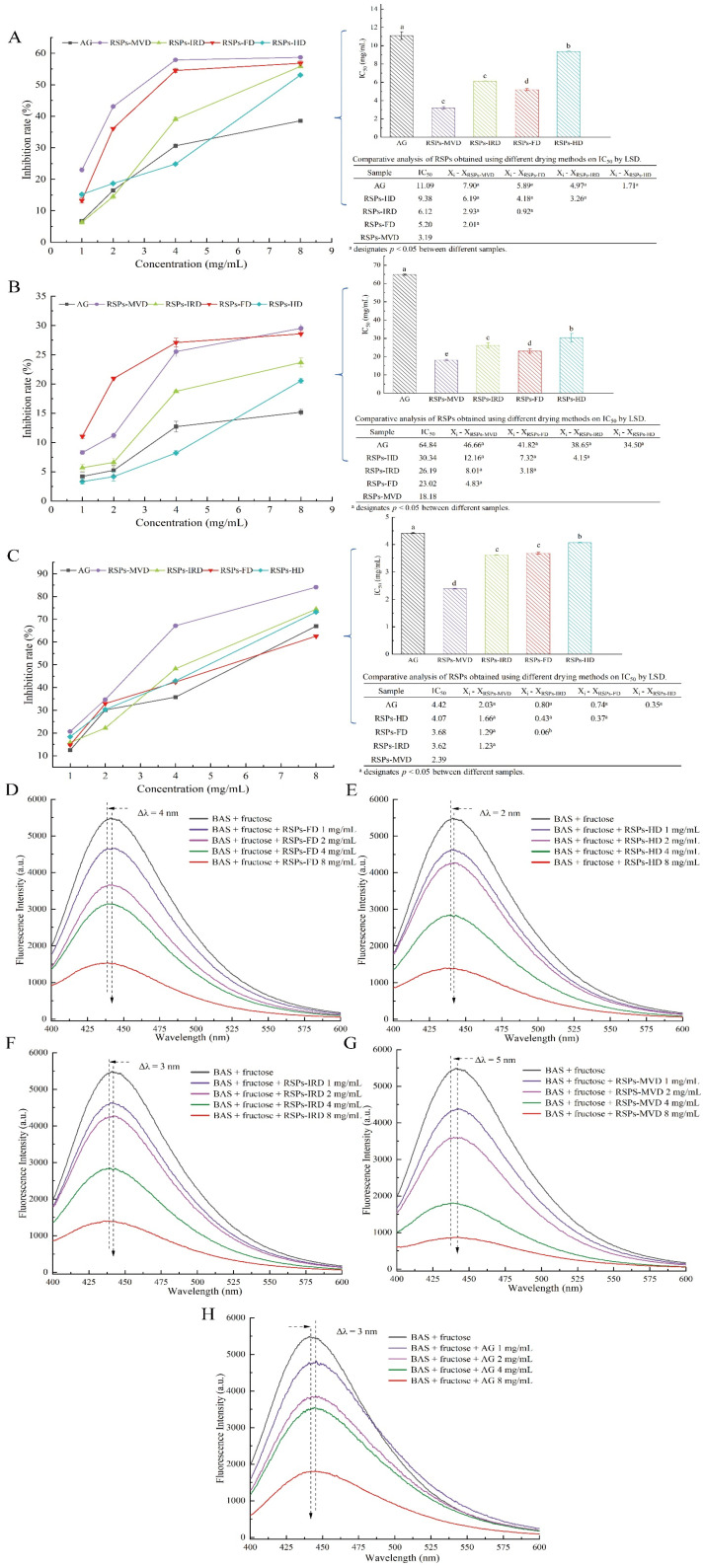
Inhibitory effects on glycation products for RSPs: (**A**) fructosamine inhibition (**B**) α-dicarbonyl compound inhibition; (**C**) AGE inhibition; (**D**–**H**) fluorescence spectra of AGEs in the presence of increasing concentrations of RSPs and AG. IC_50_ values with no common letters are significantly different (*p* < 0.05).

During the early stage of glycation, amino groups in proteins react with carbonyl groups of reducing sugars to form Schiff bases, which quickly rearrange to form more stable Amadori products, with fructosamine predominantly [62]. Amadori products can reduce nitro blue tetrazolium (NBT) in alkaline solutions, forming a colored product that absorbs maximally at 530 nm. As shown in Figure 6A, all RSPs and AG inhibited the formation of Amadori products in a dose-dependent manner. The IC_50_ values for RSPs-MVD, RSPs-FD, RSPs-IRD, RSPs-HD, and AG are 3.19, 5.20, 6.12, 9.38, and 11.09 mg/mL, respectively. These results indicate that RSPs significantly inhibit Amadori product formation, surpassing the effectiveness of AG. Among the RSPs tested, RSPs-MVD exhibited the strongest inhibition.

During the intermediate stage of glycation, Amadori products undergo further chemical changes, including dehydration and rearrangement, forming highly reactive dicarbonyl compounds such as glyoxal and acetaldehyde [63]. These were quantified using UV absorbance at 290 nm with the Girard-T assay. Figure 6B shows that all RSPs and AG decreased the generation of dicarbonyl compounds in a dose-dependent manner. The IC_50_ values were 18.18 mg/mL for RSPs-MVD, 23.02 mg/mL for RSPs-FD, 26.19 mg/mL for RSPs-IRD, 30.34 mg/mL for RSPs-HD, and 64.84 mg/mL for AG. This sequence indicates that RSPs are more effective than AG, with RSPs-MVD being the most potent.

In the late stage of glycation, reactive dicarbonyl compounds react and crosslink with free amino groups in proteins to form complex, stable, and irreversible AGEs. The inhibitory effects of RSPs on AGE formation were evaluated using fluorescence measurements [63]. Figure 6C demonstrates that RSPs inhibited AGE formation in a dose-dependent manner, with RSPs-MVD being the most effective at an IC_50_ value of 2.39 mg/mL (*p* < 0.05), compared to AG’s 4.42 mg/mL. Figure 6D–H further illustrates that RSPs and AG led to a dose-dependent decrease in fluorescence intensity, with RSPs-MVD showing the strongest quenching effect. A noticeable red shift in the maximum absorption peak indicated increased polarity around the fluorophores due to interactions with RSPs. The efficacy of RSPs at different glycation stages indicates peak antiglycation activity in the final phase of non-enzymatic glycation, marked by the formation of stable and irreversible complexes. RSPs-MVD, with its lower molecular weight and higher uronic acid content, showed superior antiglycation activity, supporting Zhu et al.’s findings [64]. Uronic acids, known for their strong chelating properties, may interfere with glycation by binding metal ions that catalyze the formation of Amadori products [65]. Additionally, the lower molecular weight of RSPs-MVD could facilitate greater accessibility and interaction with glycation intermediates, effectively preventing their progression to AGEs [66]. This study highlights the potential of RSPs, especially RSPs-MVD, as potent inhibitors of glycation. Their ability to reduce AGE formation underscores their therapeutic potential in managing diabetic complications, offering a promising strategy against such chronic conditions.

## 4. Conclusions

This study compared the impacts of various drying techniques on the physical and chemical properties and in vitro antioxidant, antiglycation, and α-glucosidase inhibitory activities of polysaccharides from *Rosa sterilis* fruits. The findings demonstrated that different drying methods influenced both the physicochemical properties and bioactivities of RSPs. The extraction yield, total sugar, uronic acid content, molar ratios of monosaccharides, molecular weight distribution, particle size, thermal stability, and microstructure of RSPs varied with the drying technique employed. RSPs-MVD, obtained through microwave vacuum drying, exhibited lower molecular weight and higher uronic acid contents, resulting in the most potent in vitro antioxidant, hypoglycemic, and antiglycation activities. These results indicate that microwave vacuum drying is a promising pre-drying method for extracting RSPs from *R. sterilis* fruits, enhancing their bioactive properties. Moreover, RSPs have potential as functional ingredients in both the food and pharmaceutical industries. Overall, these findings lay a solid scientific foundation for the industrial application of microwave vacuum drying in extracting and processing polysaccharides from *R. sterilis* fruits. Currently, research into the bioactivities of dried polysaccharides is primarily conducted through in vitro studies, highlighting the need for further exploration of their in vivo activities. Future research will focus on elucidating the mechanisms behind the potent biological activities of RSPs.

## Figures and Tables

**Table 1:** Effects of different drying methods on extraction yield, chemical compositions, and molecular weight distribution of RSPs.

Sample	RSPs-MVD	RSPs-IRD	RSPs-FD	RSPs-HD
Yield (%)	6.93 ± 0.05 ^a^	4.14 ± 0.03 ^d^	5.89 ± 0.14 ^b^	4.98 ± 0.03 ^c^
Chemical composition (%, *w*/*w*)				
Total sugars	77.07 ± 2.05 ^a^	71.05 ± 1.26 ^c^	74.13 ± 0.97 ^b^	69.65 ± 0.42 ^c^
Protein	2.78 ± 0.36 ^a^	1.35 ± 0.13 ^b^	2.59 ± 0.13 ^a^	1.49 ± 0.38 ^b^
Uronic acid	41.14 ± 0.29 ^a^	33.76 ± 0.09 ^c^	37.74 ± 1.55 ^b^	32.48 ± 1.07 ^c^
Molecular weight distribution				
*Mw* (kDa)	142.51 ± 0.25 ^c^	197.53 ± 3.72 ^a^	161.64 ± 6.19 ^b^	202.18 ± 5.06 ^a^
*Mn* (kDa)	25.64 ± 0.36 ^b^	24.48 ± 0.80 ^b^	19.31 ± 0.80 ^b^	34.64 ± 0.19 ^a^
*Mp* (kDa)	79.17 ± 0.50 ^b^	81.18 ± 0.83 ^b^	67.82 ± 0.85 ^c^	83.11 ± 0.34 ^a^
*Mw*/*Mn*	5.56 ± 0.07 ^b^	8.07 ± 035 ^a^	8.37 ± 0.03 ^a^	5.84 ± 0.18 ^b^

Data are expressed as mean ± standard deviation (n = 3). Different lowercase letters within the same row indicate significant differences among samples (*p* < 0.05).

**Table 2:** Monosaccharide compositions and degree of esterification (DE) of RSPs.

Sample	RSPs-MVD	RSPs-IRD	RSPs-FD	RSPs-HD
Monosaccharide constituents (molar ratios, mol %)				
Fucose (Fuc)	0.18 ± 0.04 ^a^	0.09 ± 0.00 ^a^	0.26 ± 0.16 ^a^	0.22 ± 0.02 ^a^
Rhamnose (Rha)	9.98 ± 0.14 ^a^	9.89 ± 0.23 ^a^	9.96 ± 0.20 ^a^	9.88 ± 0.10 ^a^
Arabinose (Ara)	5.41 ± 0.08 ^b^	4.69 ± 0.28 ^c^	6.23 ± 0.21 ^a^	5.51 ± 0.20 ^b^
Galactose (Gal)	42.61 ± 0.58 ^a^	40.60 ± 1.11 ^b^	42.19 ± 0.41 ^a^	39.93 ± 0.65 ^b^
Glucose (Glc)	6.36 ± 0.08 ^b^	6.31 ± 0.42 ^b^	6.05 ± 0.23 ^b^	9.21 ± 0.29 ^a^
Xylose (Xyl)	1.15 ± 0.32 ^a^	0.13 ± 0.05 ^b^	1.20 ± 0.12 ^a^	1.34 ± 0.25 ^a^
Mannose (Man)	0.32 ± 0.21 ^d^	8.85 ± 0.02 ^a^	1.08 ± 0.12 ^c^	4.06 ± 0.51 ^b^
Galacturonic acid (GalA)	32.54 ± 0.59 ^a^	28.13 ± 0.88 ^b^	31.72 ± 0.92 ^a^	28.50 ± 0.64 ^b^
Glucuronic acid (GlcA)	1.47 ± 0.02 ^a^	1.31 ± 0.06 ^b^	1.31 ± 0.01 ^b^	1.35 ± 0.03 ^b^
Monosaccharide ratios				
HG (%)	22.56 ± 0.59 ^a^	18.24 ± 0.65 ^b^	21.76 ± 1.11 ^a^	18.62 ± 0.65 ^b^
RG-I (%)	67.98 ± 0.50 ^a^	65.07 ± 0.36 ^b^	68.34 ± 0.53 ^a^	65.20 ± 0.72 ^b^
Linearity	0.54 ± 0.02 ^a^	0.50 ± 0.02 ^b^	0.52 ± 0.02 ^ab^	0.49 ± 0.01 ^b^
MR1	3.26 ± 0.07 ^a^	2.84 ± 0.02 ^b^	3.18 ± 0.15 ^a^	2.88 ± 0.07 ^b^
MR2	4.81 ± 0.12 ^a^	4.58 ± 0.19 ^b^	4.86 ± 0.09 ^a^	4.60 ± 0.03 ^b^
DE (%)	56.36 ± 0.37 ^b^	54.68 ± 0.16 ^c^	58.91 ± 0.58 ^a^	55.74 ± 1.35 ^bc^

Data are expressed as mean ± standard deviation (n = 3). Different lowercase letters within the same row indicate significant differences among samples (*p* < 0.05). HG/% = GalA − Rha: homogalacturonan; RG-I/% = 2Rha + Gal + Ara: rhamnogalacturonan; linearity = GalA/(Fuc + Rha + GlcA + Ara + Gal + Xyl); MR1 = GalA/Rha; MR2 = (Ara + Gal)/Rha: the average size of side chains.

**Table 3:** The particle size distribution and DSC parameters of RSPs.

Sample	RSPs-MVD	RSPs-IRD	RSPs-FD	RSPs-HD
Particle size (nm)	427.73 ± 9.01 ^c^	488.80 ± 4.43 ^a^	454.47 ± 9.79 ^b^	496.00 ± 6.14 ^a^
PDI (nm)	0.24 ± 0.01 ^b^	0.33 ± 0.04 ^a^	0.34 ± 0.06 ^a^	0.34 ± 0.03 ^a^
DSC parameters				
*Tm* (°C)	134.03 ± 15.51 ^b^	149.31 ± 0.03 ^a^	138.94 ± 10.21 ^b^	149.19 ± 1.14 ^a^
Δ*Hm* (J/g)	769.96 ± 19.18 ^a^	698.19 ± 10.93 ^b^	731.96 ± 35.78 ^ab^	528.06 ± 38.87 ^c^
*Tg* (°C)	259.11 ± 0.25 ^a^	254.96 ± 0.01 ^b^	259.98 ± 0.31 ^a^	258.81 ± 0.09 ^a^
Δ*Hg* (J/g)	25.22 ± 3.05 ^a^	15.77 ± 0.58 ^c^	18.61 ± 3.05 ^b^	14.48 ± 2.05 ^c^

Data are expressed as mean ± standard deviation (n = 3). Different lowercase letters within the same row indicate significant differences among samples (*p* < 0.05).

**Table 4:** Kinetic parameters of α-glucosidase inhibition in the presence of RSPs.

Sample	Concentration (mg/mL)	*Vmax* (ΔA405/min)	*Km* (mM)	*Ki* (mg/mL)	*Kis* (mg/mL)	*Kis*/*Ki*	*K_SV_* (M^−1^)	*Kq* (M^−1^·S^−1^)	*Ka* (M^−1^)	Quenching Type
RSPs-MVD	Control	0.86	21.70	17.44	1.44	0.08	7.40 × 10^4^	7.40 × 10^12^	1.78 × 10^3^	Static quenching
	0.5	0.35	9.45							
	2	0.10	3.46							
	6	0.08	2.77							
RSPs-IRD	Control	0.28	7.32	19.20	3.26	0.17	5.80 × 10^4^	5.80 × 10^12^	0.14 × 10^3^	Static quenching
	0.5	0.11	3.36							
	2	0.09	2.86							
	6	0.06	2.09							
RSPs-FD	Control	0.44	13.02	11.97	1.90	0.16	7.23 × 10^4^	7.23 × 10^12^	1.16 × 10^3^	Static quenching
	0.5	0.13	4.63							
	2	0.07	3.00							
	6	0.05	2.36							
RSPs-HD	Control	0.42	13.23	27.58	4.55	0.17	7.81 × 10^4^	7.81 × 10^12^	0.40 × 10^3^	Static quenching
	0.5	0.09	3.50							
	2	0.08	3.08							
	6	0.07	2.75							

## Data Availability

The original contributions presented in the study are included in the article, further inquiries can be directed to the corresponding author.

## Abbreviations

MVD	microwave vacuum drying
IRD	infrared drying
HD	hot air drying
FD	freeze-drying
HPAEC	high-performance anion-exchange chromatography
HPGPC	high-performance gel-permeation chromatography
FTIR	Fourier Transform Infrared Spectroscopy
XRD	X-ray Diffraction
DSC	differential scanning calorimeter
SEM	Scanning Electron Microscopy
DM	methyl esterification
*Mw*	weight average molecular weight
*Mn*	number average molecular weight
*Mp*	peak molecular weight
